# Supplementary material for: Early Neuroprotective Effects of Bovine Lactoferrin Associated with Hypothermia after Neonatal Brain Hypoxia-Ischemia in Rats
Source: Int J Mol Sci. 2023 Oct 25;24(21):15583. doi: 10.3390/ijms242115583 (PMC10650654; doi:10.3390/ijms242115583)
Supplement: Supplementary file 1 [file ijms-24-15583-s001.zip › ijms-2653172-supplementary.pdf]

Table S1. List of primers used for RT-qPCR.

	Forward	Reverse
**GAPDH**	GAA CAT CAT CCC TGC ATC CA	GCC AGT GAG CTT CCC GTT CA
**GFAP**	CAG ACT TTC TCC AAC CTC CAG	CTC CTG CTT CGA GTC CTT AAT G
**Glut-1**	GCA TCG TCG TTG GGA TCC T	CAA GTC TGC ATT GCC CAT GA
**GRIN2a (NMDAr2A)**	TCA CAG GCG TGT GCT CTG A	AAC TAT AGA TGC CCC TGC TGA TG
**HCAR1**	GTG TTG GCG AGG CTC TAC TT	AAC ACA CTT GGA GAC CCC AC
**HIF-1α**	TGG AAT GGA GCA GAA GAC AAT TT	GGT AAT CCA CTC TCA TCC ATT GAC T
**IL-1β**	GGC AAC TGT CCC TGA ACT CAA	GCC TCA AAG AAC AGG TCA TTC TC
**MCT-1**	GGG CGC CGC GAG ATA	GGC ATC TTA GGT GTC GAT GAA TT
**MCT-2**	GCT TCT TTC AGC AGC AGT GTG	TGA ATG CTA ATC CTA AAC CTC CAA
**MCT-4**	GCT GGC TAT GCT GTA TGG C	TTG AGA GCC AGA CCC AAG C
**Slc1a3 (GLAST)**	CAT TGG AGG GTT GCT GCA A	GCA GGG TGG CAG AAC TTG AG
**S100B**	CAT CAA CAA CGA GCT CTC TCA CTT	CAT CAC TTT GTC CAC CAC TTC CT
**TNF-α**	GAC CCT CAC ACT CAG ATC ATC TTC T	TCC GCT TGG TGG TTT GCT A
